# Prognostic Significance of Hemoglobin/Prognostic Nutritional Index and Hemoglobin/Red Blood Cell Distribution in Rectal Cancer

**DOI:** 10.5152/tjg.2022.22203

**Published:** 2023-02-01

**Authors:** Elif Tuğba Tuncel, Murtaza Parvizi, Engin Kut, Mesut Aydın, Elmas Kasap

**Affiliations:** 1 Department of Gastroenterology, Manisa State Hospital, Manisa, Turkey; 2 Department of Radiation Oncology, Manisa State Hospital, Manisa, Turkey; 3 Department of Medical Oncology, Manisa State Hospital, Manisa, Turkey; 4 Department of Gastroenterology, Yüzüncü Yıl University Faculty of Medicine, Van, Turkey; 5 Department of Gastroenterology, Celal Bayar University Faculty of Medicine, Manisa, Turkey

**Keywords:** H/PNI, H/RDW, nutritional status, prognosis, rectal cancer

## Abstract

**Background:**

We aimed to investigate the effect of hemoglobin/prognostic nutritional index and hemoglobin/red blood cell distribution, which are indicators of inflammation and nutrition, on prognosis and survival in patients with rectal cancer.

**Methods:**

The retrospective study reviewed medical records of 138 patients with rectal cancer who were followed up between 2010 and 2021. The effects of hemoglobin/red blood cell distribution, hemoglobin/prognostic nutritional index, tumor stage, and lymph node status on survival and prognosis were evaluated using univariate and multivariate analyses. Overall survival and disease-free survival were calculated for both groups.

**Results:**

Survival and prognosis were found to be significantly better in nonanemic patients with the hemoglobin/prognostic nutritional index higher than the cut-off value than in anemic patients with a normal or lower hemoglobin/prognostic nutritional index. Similarly, survival and prognosis were found to be significantly better in nonanemic patients with a hemoglobin/red blood cell distribution higher than the cut-off value than in anemic patients with a normal or lower hemoglobin/red blood cell distribution.

**Conclusion:**

The results indicated that nutrition and inflammatory markers have independent prognostic significance in rectal cancer. These markers are simple, inexpensive, and useful biomarkers commonly used in clinical practice, and they were found to predict overall survival and disease-free survival independently.

**Main Points**

- Nutrition and inflammatory markers have independent prognostic significance in rectal cancer.
- Survival and prognosis were found to be significantly better in nonanemic patients with higher hemoglobin/prognostic nutritional index.
- Similarly, survival and prognosis were found to be significantly better in nonanemic patients with higher hemoglobin/red blood cell distribution.
- These markers are simple, inexpensive, and useful biomarkers commonly used in clinical practice.

## Introduction

Colorectal cancer is a common life-threatening disease. According to the World Health Organization (WHO) GLOBOCAN 2020 database, 19.3 million new cancer cases were diagnosed and approximately 10 million cancer deaths occurred around the world in 2020. Most common cancers in men include lung, prostate, and colorectal cancers, while most common cancers in women include breast and colorectal cancers. The incidence of colon cancer (CC) is 9.8% (CC 6% + rectal cancer 3.8%), and its mortality rate is 9.2% (CC 5.8% + rectal cancer 3.4%). Colon cancer is more common in men than in women. Rectal cancers represent the second most common cancer in the large intestine after proximal CCs. Local recurrence or metastasis can be seen in approximately 20%-45% of the cases.^1,2^ This stage of the disease is the gold standard for determining the prognosis and surveillance follow-up and also for appropriate treatment selection. Knowledge of individualized prognostic factors is essential for identifying the clinical risk and predicting survival and recovery. Additionally, cancer development, progression, and prognosis correlate with patients’ inflammatory and nutritional status.^3,4^ Systemic inflammatory markers play an important role in colorectal carcinogenesis and progression.^5,6^ The prognostic nutritional index (PNI) reflects patients’ nutritional and immune status and is calculated based on the serum albumin concentration and the peripheral blood lymphocyte count.^7,8^ The prognostic significance of PNI and red blood cell distribution (RDW) has been documented in numerous malignancies such as colon, gastric, lung, head and neck, esophageal, hepatocellular, and pancreatic cancers. In addition, by combining PNI and RDW with hemoglobin, the hemoglobin-to-PNI ratio (H/PNI) and hemoglobin-to-RDW ratio (H/RDW) have been created, which show both nutritional and inflammatory activity.^9,10^ In the present study, we aimed to investigate the effects of H/PNI and H/RDW on clinical findings, survival, and prognosis in patients with rectal cancer.

## Materials and Methods

The retrospective study reviewed medical records of 138 patients with rectal cancer who received neoadjuvant chemoradiotherapy and were followed up in Manisa State Hospital between 2010 and 2021. Patients that had clinicopathologically confirmed primary rectal cancer and no other synchronous malignancy were included in the study. Patients with CC associated with inflammatory bowel disease, history of hereditary CC, malignancy in other organs, hematological malignancy, acute or chronic inflammatory disease, history of autoimmune disease, history of infection or sepsis, and a history of blood transfusion within the last 6 months were excluded from the study. Age, gender, histological classification, lymph node metastasis, distant metastasis, tumor stage, complete blood count (CBC) parameters (white blood cell count, H, lymphocyte count, monocyte count, and RDW), serum albumin, PNI, carcinoembryonic antigen, and carbohydrate antigen 19-9 values were reviewed for each patient. Tumor staging was performed using abdominal-pelvic magnetic resonance imaging. Relationship between tumor stage and overall survival (OS) and disease-free survival (DFS) was evaluated. The clinicopathological features evaluated in the study included tumor type, grade, size, localization, and presence of metastasis. Based on the WHO guidelines, anemia was defined as a hemoglobin value of <13 g/dL in adult men and as <12 g/dL in women. Accordingly, the patients were divided as anemic and nonanemic and both H/PNI and H/RDW were calculated for both groups. The effects of H/RDW, H/PNI, tumor stage, and lymph node status on survival and prognosis were evaluated using univariate and multivariate analyses. In addition, OS was compared between the 2 groups. Overall survival was defined as the time from diagnosis to death and DFS was defined as the time from the time of diagnosis to progression/relapse.

Hemoglobin-to-RDW ratio was calculated using the following formula: H (g/dL)/RDW (%).

Onodera’s PNI was calculated using the following formula: (10 × serum albumin [g/dL]) + (0.005 × absolute lymphocyte count [×10_9_/L]).^11^

The study was conducted in accordance with the Declaration of Helsinki and the study protocol was approved by Celal Bayar University Medical School Health Sciences Ethics Committee (Date: November 24, 2021; No: 2021/20.478.486/1035).

### Statistical Analysis

Data were analyzed using Statistical Package for the Social Sciences software for Windows version 21 (IBM Corp.; Armonk, NY, USA). Descriptives were expressed as median, mean, standard deviation, minimum, and maximum for continuous variables and as frequencies (n) and percentages (%) for categorical variables. Cut-off values of H/PNI and H/RDW were determined using receiver operating characteristics curve analysis. Normal distribution of variables was analyzed using visual (histogram) and analytical methods (Kolmogorov–Smirnov/Shapiro–Wilk test). Normally distributed parameters were compared using Student’s *t*-test and non-normally distributed variables were compared using Mann–Whitney *U* test. Independent groups were compared using Chi-square or Fischer’s exact test. The correlation between H/PNI and H/RDW was analyzed using Spearman’s correlation coefficient. Survival analysis was performed using the Kaplan–Meier method. Significant variables in univariate analysis were introduced into a multivariate Cox model. Clinicopathological features, prognostic value of H/RDW and H/PNI, OS, and DFS were calculated using multivariate analysis. *P* <.05 was considered significant.

## Results

**Figure 1.**
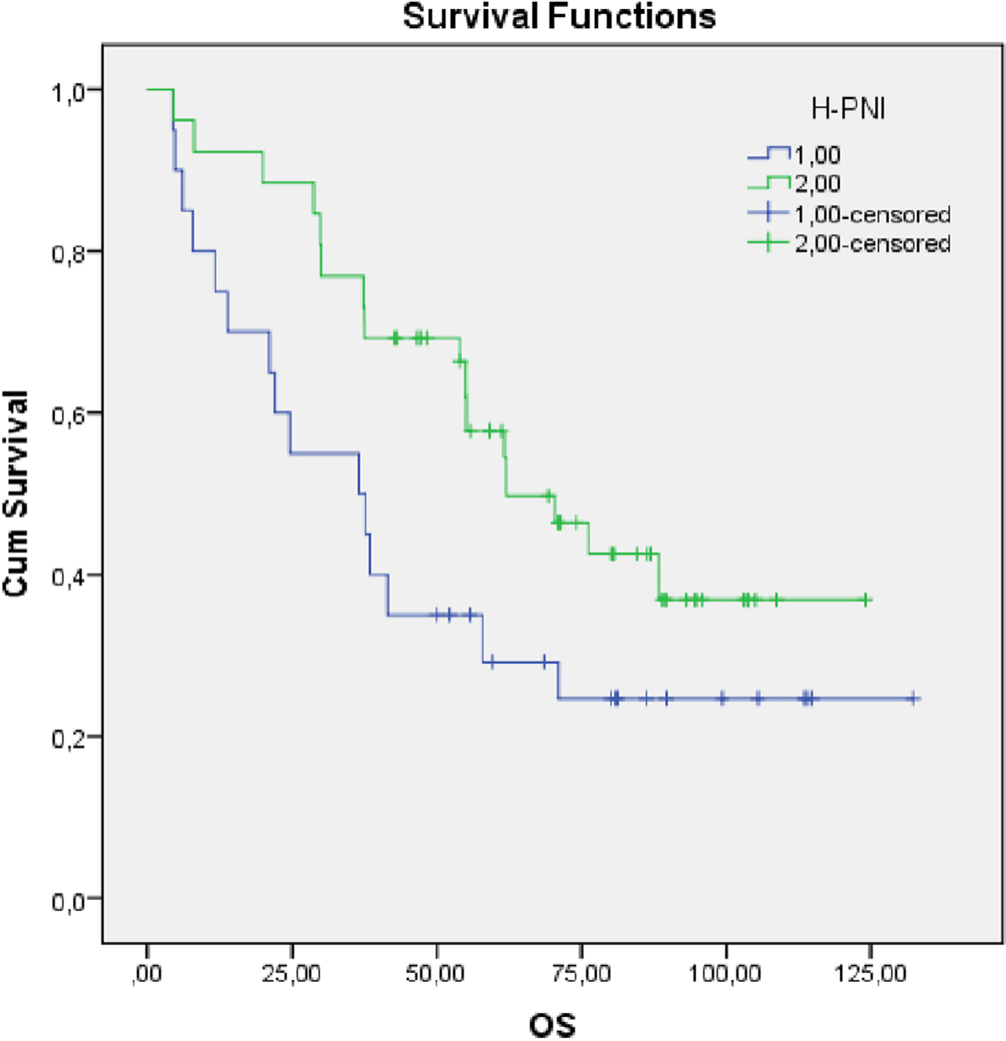
Kaplan–Meier analysis of the relationship between H/PNI and OS (high H/PNİ score (≥55.25) = 2.00-censored, low H/PNİ score (<55.25) = 1.00-censored). OS, overall survival; H/PNI, hemoglobin-to-prognostic nutritional index ratio.

**Figure 2.**
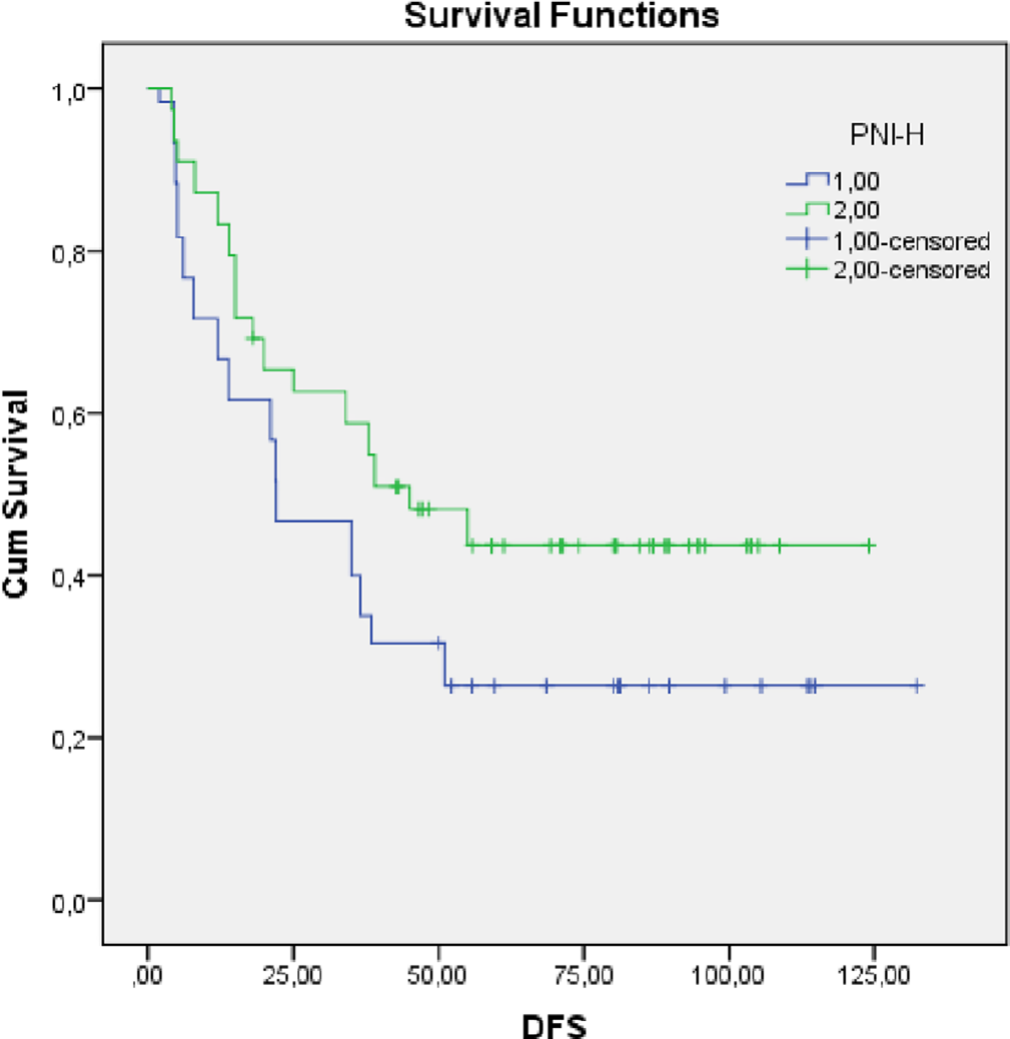
Kaplan–Meier analysis of the relationship between H/PNI and DFS (high H/PNİ score (≥55.25) = 2.00-censored, low H/PNİ score (<55.25) = 1.00-censored). DFS, disease-free survival; H/PNI, hemoglobin-to-prognostic nutritional index ratio.

**Figure 3.**
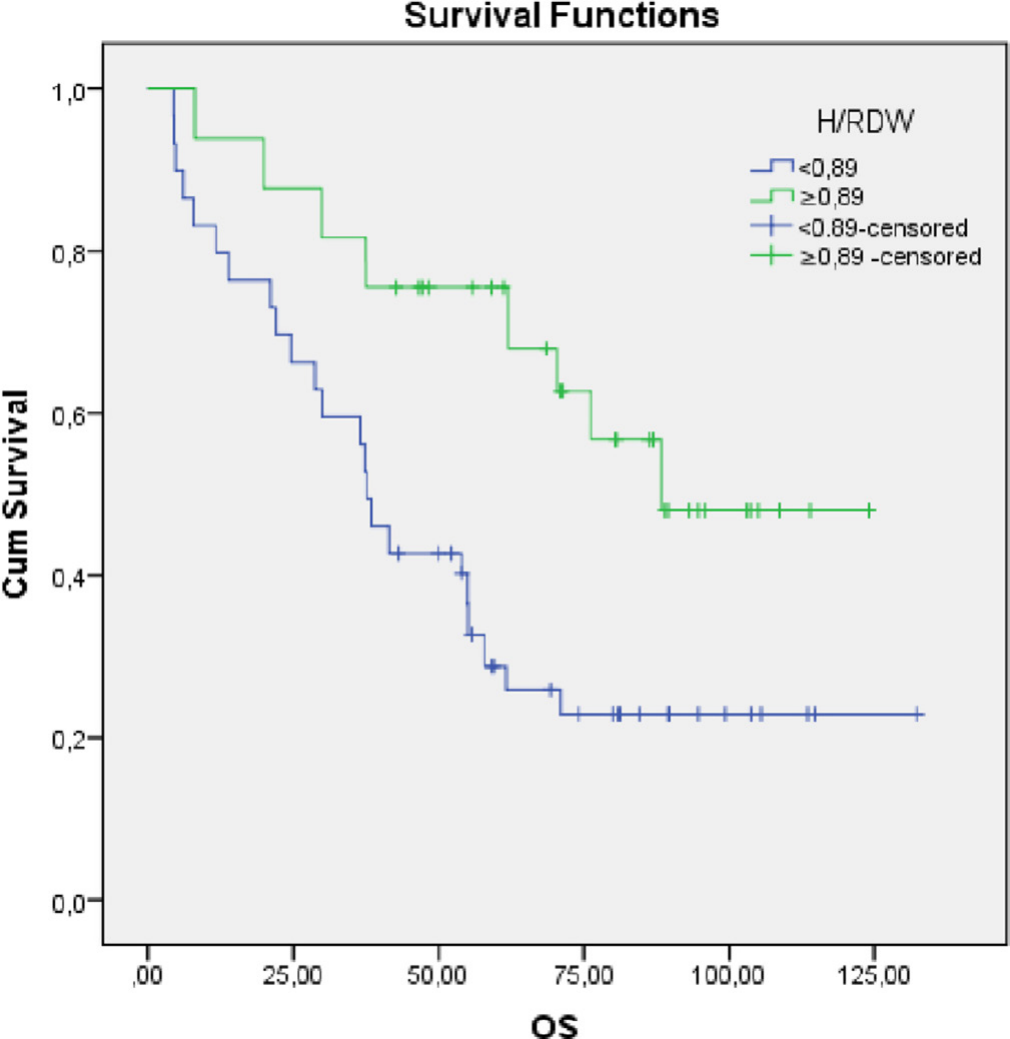
Kaplan–Meier analysis of the relationship between H/RDW and OS (high H/RDW score ≥0.89, low H/RDW score ≤0.89). OS, overall survival; H/RDW, hemoglobin-to-red blood cell distribution ratio.

**Figure 4.**
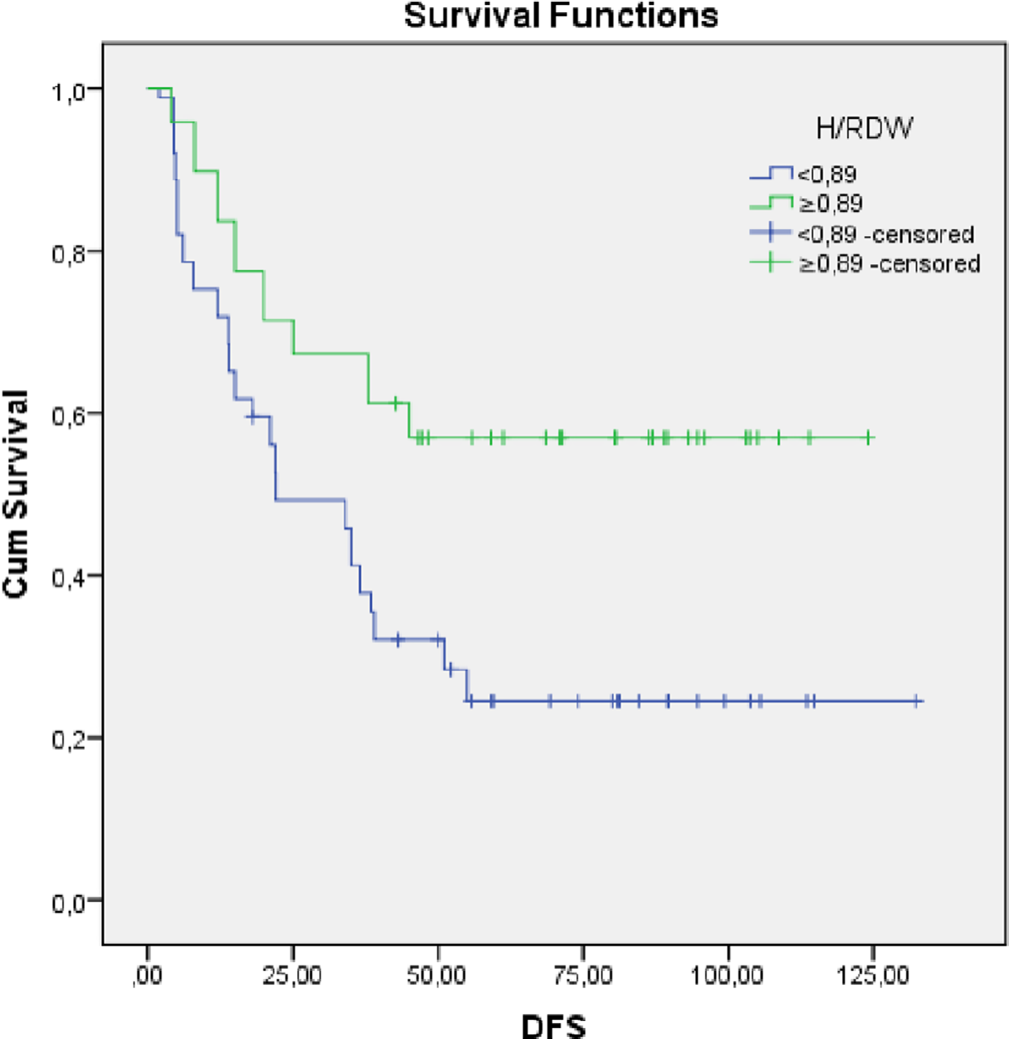
Kaplan–Meier analysis of the relationship between H/RDW and DFS (high H/RDW score ≥0.89, low H/RDW score ≤0.89). DFS, disease-free survival; H/RDW, hemoglobin-to-red blood cell distribution ratio.

The 138 patients comprised 83 (60.1%) men and 55 (39.9%) women with a mean age of 62 (range: 28-84) years. The median follow-up was 40 months, and the median survival was 55.06 months (95% CI: 40.33-70.10). No surgical treatment was performed in 17 (13.3%) patients. One-year survival rate was 91.30%, 3-year survival rate was 73.91%, and 5-year survival rate was 57.25%. Based on clinical, radiological, and pathological findings, all the patients were confirmed to have a stage III tumor. Anemia was detected in 68 (49.2%) patients (Table 1). Mean OS was 38.47 (range: 34-42.9) months in anemic patients as opposed to 61.93 (range: 42.43-81.42) months in nonanemic patients (*P *= .44). The cut-off values for H/PNI and H/RDW were 55.25 and 0.89, respectively. Accordingly, patients were divided as patients with high H/RDW (≥0.89) and low H/RDW (<0.89) and as patients with high H/PNI (≥55.25) and low H/PNI (<55.25) (Table 2). Hemoglobin-to-prognostic nutritional index ratio and H/RDW were found to be associated with prognosis in both univariate and multivariate analyses. Survival and prognosis were found to be significantly better in nonanemic patients with a H/PNI higher than the cut-off value than in anemic patients with a normal or lower H/PNI (*P *< .005). Similarly, survival and prognosis were found to be significantly better in nonanemic patients with a H/RDW higher than the cut-off value than in anemic patients with a normal or lower H/RDW (*P *< .05). In both univariate and multivariate analyses, H/RDW and H/PNI were found to be independent predictors of OS and DFS (Table 3) (Figures 1, 2, 3, and 4).

## Discussion

Prognostic values of inflammatory and nutritional parameters have been investigated by numerous studies. Although these parameters are important prognostic factors in cancer patients, an analysis of a single parameter may not provide sufficient predictive power in clinical practice and thus an analysis of multiple parameters is essential. For this reason, the present study analyzed the effects of nutritional and inflammatory markers (H/RDW and H/PNI) on cancer-specific survival and also evaluated their prognostic values in patients with rectal cancer.

Inflammation and nutritional status have a significant effect on disease progression and survival in CC.^12^ Local inflammation in the tumor microenvironment is indicative of systemic inflammatory response syndrome (SIRS). Numerous recent studies investigating the role of SIRS in cancer progression and prognosis have shown that both cancer progression and prognosis correlate with inflammatory and nutritional status except for pathological features.^13^ The relationship between inflammation and neoplasia was first demonstrated by Virchow in 1863, after observing the presence of leukocytes in neoplastic tissue. Immunomodulatory cytokines and systemic inflammatory markers (neutrophils, lymphocytes, interleukin-1, 6, 8, 9, and tumor necrosis factor-alpha) in the tumor microenvironment play an important role in tumor formation, invasion, and metastasis. In cancer patients, malnutrition is typically caused by high metabolic rate, systemic inflammation, anorexia, and hypoalbuminemia. In patients with CC, malnutrition is closely associated with mortality, with a reported prevalence of 33%-41%. In chronic malnutrition, cytokine response and subsequent immune system activation are impaired. As cancer progresses, chronic malnutrition and systemic inflammatory response against the tumor suppress hemoglobin and albumin synthesis. Additionally, anemia and malnutrition reduce patients’ quality of life, treatment response, and cancer survival.^14^ Complete blood count parameters such as hemoglobin, leukocyte, thrombocyte, and RDW have been investigated as prognostic factors in various malignancies. Peripheral blood cells are indicators of the inflammatory and immune response against the tumor and have independent prognostic significance. The presence of anemia before treatment has been shown to be a poor prognostic factor in nasopharyngeal, head and neck, cervical, esophageal squamous cell cancer, and gastrointestinal cancers.^15^ Inflammation and nutrition-related parameters (neutrophil-to-lymphocyte ratio [NLR], platelet-to-lymphocyte ratio, lymphocyte-to-monocyte ratio, PNI, and Glasgow prognostic score) have been shown to be associated with survival in numerous solid cancers including gastric cancer, non-small cell lung cancer (NSCLC), ovarian cancer, cholangiocarcinoma, hepatocellular carcinoma, pancreatic cancer, colorectal cancer, nasopharyngeal cancer, and malignant melanoma.^16,17^ Red blood cell distribution and H are important CBC parameters commonly used as markers of anemia. Red blood cell distribution is a simple measure of red blood cell size heterogeneity. On the other hand, anemia is detected in approximately 30% of cancer patients. In recent reports, the prognostic significance of RDW has been demonstrated in numerous malignant and nonmalignant diseases. Moreover, previous studies showed that RDW was closely associated with inflammatory bowel disease activity, inflammatory status in hepatitis B patients, acute pancreatitis, and cardiovascular disease risk.^18-21^ High RDW and low hemoglobin levels have been shown to be associated with systemic inflammation and malnutrition. Anemic hypoxia affects tumor metabolism by overexpressing hypoxia-inducible factor-1, vascular endothelial growth factor, glucose transporter, and epidermal growth factor. The presence of low hemoglobin before treatment is an indicator of poor prognosis in patients with gastric cancer, esophageal squamous cell carcinoma, and cervical and nasopharyngeal cancers. Increased RDW has been found to be a poor prognostic indicator for gastric, lung, esophageal, and colon cancers. Warwick et al^22^ suggested that increased RDW is strongly associated with long-term survival in patients with NSCLC. Zhai et al^23^ showed that the H/RDW has independent prognostic significance in patients with metastatic gastric cancer. Chen et al^24^ found that RDW, NLR, and H/RDW are independent prognostic factors in patients with NSCLC. Sun et al^25^ showed that H/RDW is associated with clinical findings and prognosis in patients with esophageal squamous cell cancer. In another study, low H/RDW was found to be an indicator of aggressive tumor behavior and advanced tumor stage. As consistent with the literature, our study showed that H/RDW is an independent prognostic factor for survival and prognosis in patients with rectal cancer.

The prognostic nutritional index, calculated based on the serum albumin concentration and the peripheral blood lymphocyte count, reflects both the nutritional and immune status of the patients. The albumin produced by hepatocytes is associated with nutritional status and increased inflammatory response to the tumor. Some of the proinflammatory cytokines available in the tumor microenvironment reduce albumin synthesis. Peripheral lymphocytes, which play an important role in the immune response against the tumor, are known to reflect the immune–nutritional status of patients. Recent studies have shown that low PNI is associated with poor prognosis in numerous gastrointestinal malignancies. The cut-off values determined for PNI in CC show wide variation, ranging from 40 to 55. This wide variation is due to the differences among the studies with regard to patient numbers and techniques. Some Japanese authors determined the PNI cut-off value as 40 in CC. In our study, the cut-off value of PNI was determined as 55.25. A study by Hong et al^26^ evaluated patients with lung cancer and showed that low PNI is an independent poor prognostic factor and that the patients had low OS. Similarly, Ishizuka et al^27^ showed that survival was better in patients with postoperative PNI >45 after total gastrectomy, Jian-hui et al^28^ showed that low PNI is associated with poor prognosis in patients with stage 2-3 CC, and Jiang et al^29^ suggested that low PNI is associated with poor prognosis in patients with advanced gastric cancer. Wang et al^30^ showed that H/PNI has independent prognostic significance in patients with esophageal squamous cell cancer. In our study, in line with the literature, high H/PNI was found to be associated with a good prognosis in patients with rectal cancer.

Our study was limited since it was a retrospective and single-center study and thus had a small patient population. Accordingly, multicenter studies with larger populations are needed. On the other hand, our study is likely to contribute to the literature since previous studies have investigated individual prognostic values of H, RDW, and PNI, and our study, for the first time in the literature, evaluated the prognostic significance of H/PNI and H/RDW in malignant tumors.

## Conclusion

The results indicated that nutrition and inflammatory markers have independent prognostic significance in rectal cancer. These markers are simple, inexpensive, and useful biomarkers commonly used in clinical practice. They were found to predict OS and DFS independently. Based on our findings, we suggest that the nutritional status of patients should be improved with nutritional support prior to treatment in order to improve their quality of life, life expectancy, and prognosis in the follow-up period.

## Figures and Tables

**Table 1.** Demographic and Clinicopathological Characteristics

**Parameter**		
Age, years (median [min-max])		62 (28-82)
Gender	Male	83 (60.1%)
	Female	55 (39.9%)
PNI (median [min-max])		47.55 (30.5-83)
PNI	High	78 (56.6%)
	Low	60 (43.5%)
RDW (%) (median [min-max])		14.3 (13.4-32.44)
H/RDW	<0.89	89 (64%)
	≥0.89	49 (35.5%)
Lymphocyte (/μL) (median [min-max])		1.56 (0.08-7.4)
Monocyte (/μL) (median [min-max])		0.47 (0.15-1.5)
Hemoglobin (g/dL) mean ± SD		12.32 ± 1.59
Albumin (g/dL) mean ± SD		3.9 (2.6-5)
CEA (ng/mL) mean ± SD		4.04 (1-109)
CA 19-9 (ng/mL) mean ± SD		13.66 (1-700)
Prior surgery	None	17 (13.32%)
	Miles	46 (33.33%)
	LAR	75 (54.35%)
LVI	Positive	23 (13.8%)
Perineural invasion (PNI)	Positive	14 (10.1%)

CEA, carcinoembryonic antigen; CA19-9, carbohydrate antigen 19-9; RDW, red blood cell distribution; H/RDW, hemoglobin-to-RDW ratio; PNI, prognostic nutritional index; H/PNI, hemoglobin-to-PNI ratio; LAR, low anterior resection; LVI, lymphovascular invasion; SD, standard deviation.

**Table 2.** Univariate and Multivariate Analysis of Overall Survival

	Univariate Analysis (HR, 95% CI)	*P*	Multivariate Analysis (HR, 95% CI)	*P*
Age (years)	1.04 (1.02-1.06)	**<.001**	1.03 (1.01-1.05)	.008
Gender	1.34 (0.88-2.06)	.17		
Hemoglobin (g/dL)	−0.9 (0.79-1.03)	.34		
Albumin (g/dL)	−0.59 (0.39-0.89)	.13		
Lymphocyte (/μL)	−0.76 (0.58-1.08)	.57		
Platelet (/μL)	1.01 (0.8-1.01)	.17		
WBC (/μL)	1.06 (0.99-1.14)	.11		
Neutrophil (/μL)	1.07 (1.0-1.15)	.49		
CEA (ng/mL)	1.00 (0.98-1.02)	.95		
CA19-9 (ng/mL)	0.99 (0.99-1.03)	.53		
H/RDW	2.67 (1.62-4.38)	**<.001**	2.53 (1.38-4.63)	**.003**
H/PNI	1.90 (1.81-4.78)	**<.001**	2.83 (1.63-4.90)	**.001**
RDW (%)	2.04 (1.33-3.15)	**.001**	1.47 (0.83-2.59)	.19
Prior surgery	1.87 (1.14-2.75)	.011	1.67 (.65-4.32)	.29
LVI	2.62 (1.71-4.03)	**<.001**	1.49 (.41-5.38)	.54
PNI	2.60(1.73-4.10)	**<.001**	1.82 (.35-5.97)	.32

CEA, carcinoembryonic antigen; CA19-9, carbohydrate antigen 19-9; RDW, red blood cell distribution; H/RDW, hemoglobin-to-RDW ratio; PNI, prognostic nutritional index; H/PNI, hemoglobin-to-PNI ratio; HR, hazard ratio.

**Table 3.** Univariate and Multivariate Analysis of Disease-Free Survival

	Univariate Analysis (HR, 95% CI)	*P*	Multivariate Analysis (HR, 95% CI)	*P*
Age (years)	1.24 (1.01-1.04)	.008		
Gender	1.27 (0.83-1.94)	.279		
Hemoglobin (g/dL)	−0.91 (0.80-1.03)	.13		
Albumin (g/dL)	−0.69 (0.46-1.02)	.65		
Lymphocyte (/μL)	−0.79 (0.8-1.045)	.98		
Platelet (/μL)	1.02 (1.00-1.004)	.03	0.94 (0.55-1.61)	.82
WBC (/μL)	1.07 (0.10-1.14)	.12		
Neutrophil (/μL)	1.07 (1.0-1.15)	.61		
CEA (ng/mL)	0.99 (0.98-1.02)	.71		
CA19-9 (ng/mL)	1.01 (1.00-1.03)	.006	1.01 (1.00-1.02)	.96
**H/RDW**	**2.30 (1.40-3.77)**	**.001**	**2.36 (1.32-4.21)**	**.004**
**H/PNI**	**1.90 (1.81-4.78)**	**.001**	**1.59 (1.04-3.43)**	**.045**
RDW (%)	1.02 (0.96-1.07)	.42		
Prior surgery	2.23 (1.37-3.59)	.001	1.02 (0.68-2.14)	.97
LVI	2.38 (1.55-3.69)	.001	1.44 (0.44-4.76)	.54
PNI	2.45 (1.58-3.79)	.001	2.02 (0.54-2.56)	.295

CEA, carcinoembryonic antigen; CA19-9, carbohydrate antigen 19-9; RDW, red blood cell distribution; H/RDW, hemoglobin-to-RDW ratio; PNI, prognostic nutritional index; H/PNI, hemoglobin-to-PNI ratio; HR, hazard ratio.
